# A Rare Case of Obstructive Jaundice Secondary to Hemobilia Post Endoscopic Ultrasound Guided Liver Biopsy

**DOI:** 10.1002/deo2.70305

**Published:** 2026-02-25

**Authors:** Dhruv Patel, Anas Khouri, Ali Totonchian, Asad Ur Rahman

**Affiliations:** ^1^ Current Affiliations: Medical College Baroda India; ^2^ Present address: Department of Gastroenterology Cleveland Clinic Weston Florida USA; ^3^ Present address: Current Affiliations: University of New Mexico Albuquerque New Mexico USA

**Keywords:** ERCP, EUS‐guided liver biopsy, Hemobilia, Obstructive jaundice

## Abstract

Hemobilia is an uncommon complication of endoscopic liver biopsy (LB), rarely resulting in clinically significant biliary obstruction. We describe the second reported case of hemobilia‐induced obstructive jaundice secondary to endoscopic ultrasound‐guided LB (EUS‐LB). A 57‐year‐old man presented with jaundice and abdominal pain 2 days after undergoing EUS‐LB for persistently high liver enzymes, which revealed nonspecific biliary dilation. Endoscopic retrograde cholangiopancreatography (ERCP) revealed hemobilia‐induced biliary blockage, relieved with sphincterotomy and stent placement, suggestive of sphincter of Oddi dysfunction and papillary stenosis as the initial cause of liver enzyme elevation. This case emphasizes the significance of considering hemobilia as a differential for obstructive jaundice post EUS‐LB, which is generally regarded as a safe and minimally invasive procedure.

**Trial Registration**: Not applicable.

## 1 Introduction

Hemobilia is a rare complication of liver biopsy (LB), with an incidence rate of less than 1% in percutaneous liver biopsies [1, 2]. Endoscopic ultrasound‐guided LB (EUS‐LB) has emerged as a safer alternative to percutaneous LB (PC‐LB) and transjugular LB (TJ‐LB) approaches, with a lower incidence of complications such as vascular injury and clinically significant hemobilia [3]. This fact is largely due to real‐time visualization of hepatic and peri‐hepatic structures, allowing for the avoidance of major vessels and bile ducts during needle advancement. The trans‐gastric or trans‐duodenal approach used in EUS‐LB provides stable, controlled access to the liver and minimizes the risk of injuring large intrahepatic vessels ‐an inherent limitation of the percutaneous technique, which requires traversing the abdominal wall and liver capsule. Moreover, the use of fine needle biopsy (FNB) needles, particularly the 19‐gauge variety, has been associated with a lower rate of bleeding and other adverse events compared to the larger‐bore needles commonly employed in PC‐LB [3]. EUS‐LB has a favorable safety profile, yet rare complications such as hemobilia can occur. To date, only one case of hemobilia presenting as obstructive jaundice after EUS‐LB has been reported [4]. We present the second documented case, occurring in the setting of sphincter of Oddi dysfunction (SOD) type 1, and discuss potential risk factors associated with this presentation.

## 2 Case Presentation

**FIGURE 1:**
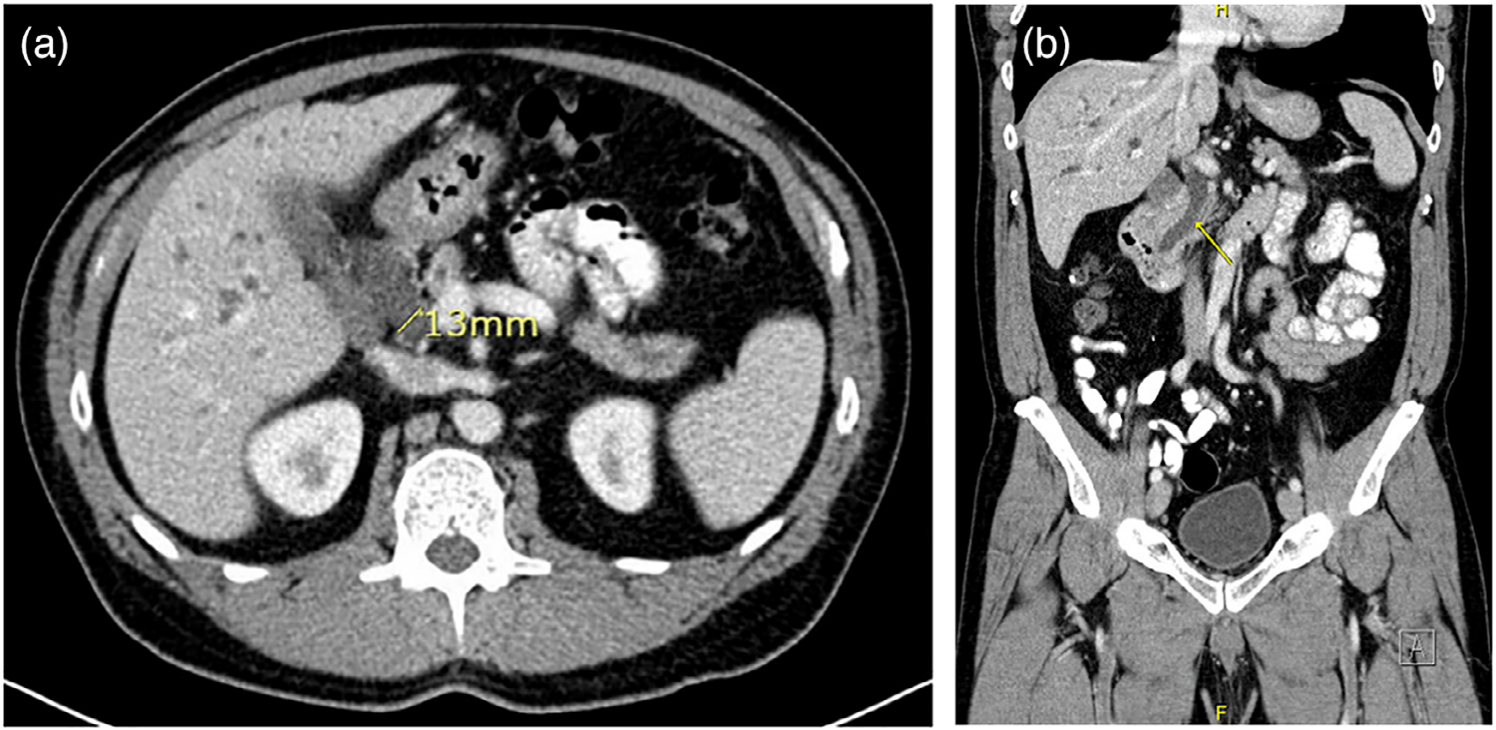
Contrast‐enhanced computed tomography (CT) abdomen with (a) cross‐sectional and (b) coronal views demonstrating significant common bile duct dilatation (>13 mm).

A 57‐year‐old asymptomatic male was referred for evaluation of chronically elevated liver enzymes for 2 years, with AST of 106 U/L, ALT of 351 U/L, ALP of 94 U/L, and T. bilirubin of 1 mg/dL. The patient was not on any antiplatelet or anticoagulant therapy, with baseline laboratory results indicating a hemoglobin level of 15 g/dL, a platelet count of 252 k/µL, and a PT (INR) ratio of 1. He had no metabolic syndrome or alcohol use, and his chronically elevated liver enzymes workup was negative for acute hepatitis panel, anti‐nuclear antibodies, anti‐smooth muscle antibodies, anti‐mitochondrial antibodies, and normal immunoglobulin G level. Right upper quadrant ultrasound (RUQ‐US) and contrast‐enhanced computed tomography (CE‐CT) demonstrated normal liver morphology without focal hepatic lesions. The same contrast‐enhanced CT images (Figure 1a,b) demonstrated persistent intra‐ and extra‐hepatic biliary ductal dilatation, with the common bile duct measuring greater than 1.3 cm in diameter (normal <0.7 cm).

Because of the hepatocellular pattern of liver enzyme elevation and the inability to fully exclude biliary stones or strictures, a shared decision was made with the patient to proceed with EUS to evaluate the biliary tree. In addition, EUS‐guided fine‐needle biopsy of the liver was planned to assess for intrinsic hepatic or biliary pathology, including autoimmune hepatitis, primary biliary cholangitis, primary sclerosing cholangitis, autoimmune cholangitis, or overlap syndromes.

The EUS‐guided approach to LB was selected instead of a percutaneous approach to minimize the number of procedures, as the patient required EUS regardless. This technique is generally better tolerated, associated with less post‐procedural pain and a shorter recovery time, and offers a lower risk of adverse events due to real‐time visualization of vascular structures with Doppler.

An EUS‐LB was performed using a 19‐gauge Acquire needle (Boston Scientific) with the wet‐heparin technique. Appropriate biopsy sites free of intervening vessels or ducts were identified. A single transgastric pass was made into the left hepatic lobe and a single transduodenal pass into the right hepatic lobe, with three actuations performed during each pass. The needle was withdrawn in a zig‐zag fashion, and Doppler imaging was used to confirm the absence of bleeding along the needle tract.

**FIGURE 2:**
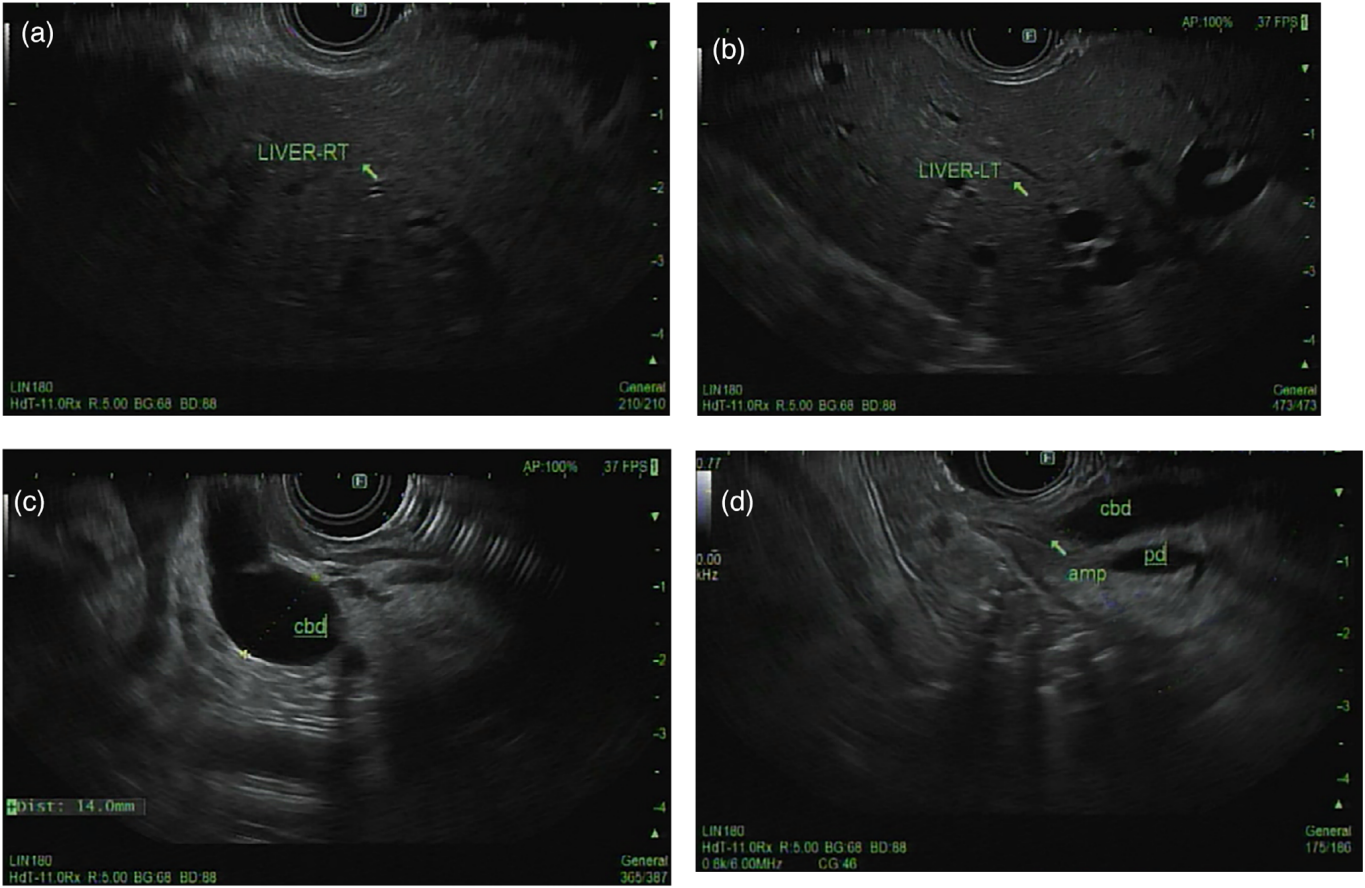
Endoscopic ultrasound (EUS) prior to biopsy showing normal Right (a) and Left (b) liver lobe parenchyma with dilated biliary tree and common bile duct (CBD) > 13 mm and visible ampulla (c, d).

The EUS imaging done prior to biopsy showed results consistent with previous imaging findings, with no stones or strictures in the dilated biliary tree [Figure 2a–d]. Biopsy further showed nonspecific areas of dilated bile ducts with mild portal chronic inflammation. The trichrome stain showed periportal fibrosis with no bridging fibrosis. No steatosis reported, and the copper stain and alpha 1 anti‐trypsin globules were negative. Two days post‐biopsy, he presented back with abdominal pain and jaundice. He had elevated liver enzymes with bilirubin of 37 mg/dL. Hemoglobin dropped to 11.8 g/dL from a baseline of 15 g/dL, and the platelets dropped to 138 k/µL from a baseline of 252 k/µL. Both RUQ and CT angiography (CTA) were performed and showed stable intrahepatic and extrahepatic biliary ductal dilatation, which was unchanged compared to the previous US and CT. There was no active contrast extravasation visualized on the CTA but there was subtle intraductal heterogeneity. Although hemobilia is an exceedingly rare complication after EUS‐LB because the puncture is performed under direct Doppler visualization, his presentation, only 2 days post‐biopsy, along with the decrease in hemoglobin and platelets, raised the suspicion of hemobilia.

**FIGURE 3:**
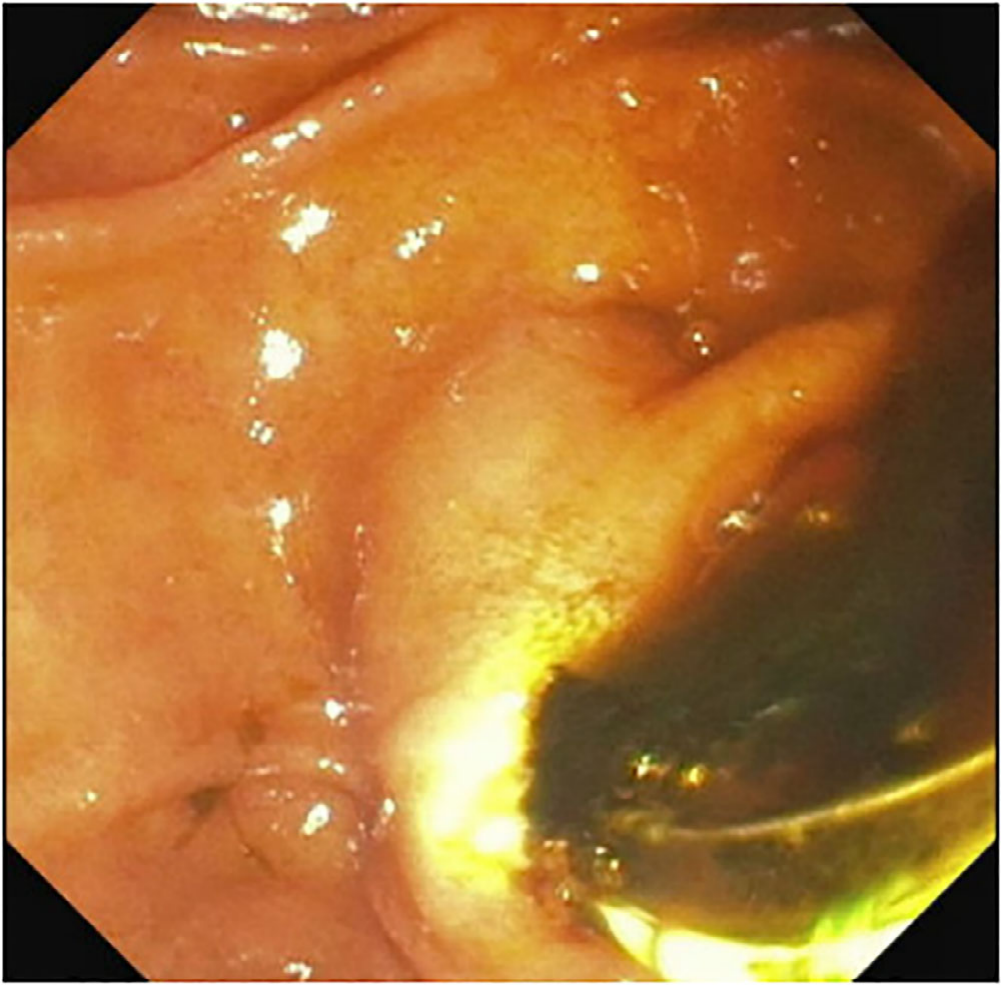
Endoscopic retrograde cholangiopancreatography (ERCP) view showing blood clot drainage from the ampulla of Vater probed with ERCP.

**FIGURE 4:**
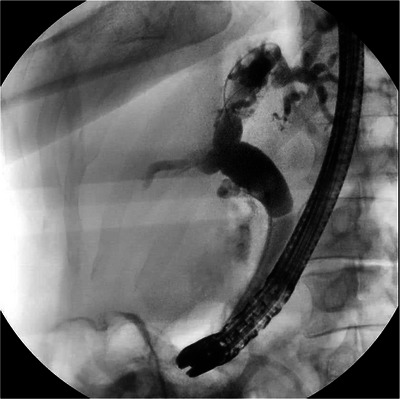
Cholangiogram immediately post‐clot drainage showing biliary tree dilation with no filling defects, strictures, or obstructions.

Endoscopic retrograde cholangiopancreatography (ERCP) was done, and it revealed a clot from hemobilia occluding the biliary tree, causing acute obstructive jaundice [Figure 3]. Sphincterotomy and balloon sweep cleared the biliary tree, and a plastic stent was placed. On cholangiogram, the entire biliary tree was dilated with no filling defects, strictures, or obstructions in the biliary tree [Figure 4]. A few days post‐procedure, symptoms resolved, and liver enzymes normalized. After ruling out primary sclerosing cholangitis, primary biliary cholangitis, and autoimmune cholangitis based on cholangiogram, histology, and lab findings, the patient was diagnosed with papillary stenosis causing SOD type 1 as the underlying cause of the original elevated liver enzymes and biliary dilation, both of which improved following sphincterotomy and stent placement.

## 3 Discussion

Hemobilia is a rare complication of LB. In one study, only four cases were reported among 68,276 PC‐LBs, with a rate of 0.00005% [1]. It is relatively common in PC‐LB and transjugular approaches but rarely seen in cases of EUS‐LB, owing to real‐time imaging and avoidance of major vascular or biliary structures [3]. Nevertheless, hemobilia can still occur, but presentation as obstructive jaundice is exceedingly rare [4]. Obstructive jaundice due to hemobilia after EUS‐LB has been reported only once previously [4]. In that case, Kawakubo et al. described a patient with hilar cholangiocarcinoma who developed abdominal pain and scleral icterus 4 days post‐biopsy with T. bilirubin levels of 6.6 mg/dL. A 5‐Fr endoscopic nasobiliary drain was placed for a total of 8 days with resolution of the patient's symptoms. In contrast, our patient presented within 48 h of the EUS‐guided biopsy for further evaluation of biliary duct changes of unknown significance, with a T. bilirubin of 37 mg/dL managed with endoscopic sphincterotomy and stent placement. A shared feature in both cases is the presence of a narrowed biliary tract, which likely contributed to clot retention and subsequent obstruction, highlighting an under‐recognized risk factor.

Although hemobilia after EUS‐LB is quite rare, evidence from PC‐LB literature identifies several procedure‐related factors that may influence bleeding risk. Larger‐caliber and cutting‐type needles have been associated with higher bleeding rates in large series of ultrasound‐guided percutaneous biopsies, whereas the impact of the number of passes or actuations remains inconsistent [5, 6]. Because robust data specific to EUS‐LB are nonexistent, these percutaneous findings are often extrapolated when assessing risk. In our patient, even though Doppler guidance was used and an appropriate technique was followed, these procedural factors combined with underlying papillary stenosis may have allowed minor bleeding to progress to clinically significant hemobilia and clot retention.

Hemobilia can occur anytime within 10 days of the biopsy [7]. ERCP is both diagnostic and therapeutic in most cases, with sphincterotomy and stent placement to relieve biliary obstruction [8]. In case of persistent bleeding, CT angiography can be important in localizing the bleeding site, and treatment is mainly trans‐catheter approach‐based arterial embolization [9]. For instance, Ion et al. reported a case of massive hemobilia following percutaneous cholecystectomy, where angiography revealed a biliary‐arterial fistula in segment IV of the liver due to iatrogenic injury, which was successfully treated with embolization using metallic coils. When angiography is not feasible or ineffective, alternative options such as selective arterial ligation or hepatectomy may be considered [9, 10].

This case suggests that although minor hemobilia after EUS‐LB is typically insignificant and goes unnoticed, it can become clinically significant when superimposed on pre‐existing biliary narrowing, leading to clot formation and obstructive jaundice. This highlights the need to exercise caution and monitoring for this potential complication post‐EUS‐LB.

## Author Contributions

**Dhruv Patel**: conceptualization, literature review, and manuscript drafting. **Anas Khouri**: data aquisition and interpretation, critical ‐revision, and supervision. **Ali Totonchian**: manuscript revision and refining. **Asad Ur Rahman**: final approval of the version to be published and overall guidance.

## Funding

The authors received no financial support for the research, authorship, and/or publication of this article.

## Ethics Statement

Ethics approval was not required for this case report in accordance with institutional policies.

## Consent

Patient consented.

## Conflicts of Interest

The authors declare no conflicts of interest.

## References

[1] F. Piccinino , E. Sagnelli , G. Pasquale , and G. Giusti , “Complications Following Percutaneous Liver Biopsy: A Multicentre Retrospective Study on 68,276 Biopsies,” Journal of Hepatology 2, no. 2 (1986): 165–173. 3958472 10.1016/s0168-8278(86)80075-7

[2] M. Ramchandani , E. van Sonnenberg , A. Mujoomdar , and K. Tuncali , “Delayed Symptomatic Hemobilia After Ultrasound‐guided Liver Biopsy: A Case Report,” AJR American Journal of Roentgenology 179, no. 1 (2002): 275–277.12076951

[3] A. Sarkar , K. Das , Shalimar , et al., “Endoscopic Ultrasound‐guided Liver Biopsy in Clinical Practice,” Gastro Hepatol Advances 1, no. 6 (2022): 936–941. 10.1016/j.gastha.2022.07.007 PMC11308829 39131258

[4] K. Kawakubo , H. Isayama , N. Takahara , et al., “Hemobilia as a Rare Complication After Endoscopic Ultrasound‐guided Fine‐needle Aspiration for Hilar Cholangiocarcinoma,” Endoscopy 43, no. Suppl 2 UCTN (2011): E175–E176. 22020713 10.1055/s-0030-1256783

[5] M. Mueller , W. Kratzer , S. Oeztuerk , M. Wilhelm , R. A. Mason , and M. M. Haenle , “Percutaneous Ultrasonographically Guided Liver Punctures: An Analysis of 1,961 Patients Over a Period of Ten Years,” BMC Gastroenterology [Electronic Resource] 12 (2012): 173, 10.1186/1471-230X-12-173. 23216751 PMC3552862

[6] M. Midia , D. Odedra , A. Shuster , R. Midia , and J. Muir , “Predictors of Bleeding Complications Following Percutaneous Image‐guided Liver Biopsy: A Scoping Review,” Diagnostic and Interventional Radiology 25, no. 1 (2019): 71–80, 10.5152/dir.2018.17525. 30644369 PMC6339629

[7] N. O. Machado , “Hemobilia Post Liver Biopsy: Mechanism, Presentation, Complications and Management,” Journal of Gastroenterology Pancreatology and Liver Disorders 2, no. 3 (2015): 1–14.

[8] L. J. Worobetz , E. A. Shaffer , J. K. Kelly , S. J. Urbanski , and N. B. Hershfield , “Hemobilia After Percutaneous Liver Biopsy: Role of Endoscopic Retrograde Cholangiopancreatography and Sphincterotomy,” American Journal of Gastroenterology 78, no. 3 (1983): 182–184. 6402924

[9] T. Marynissen , G. Maleux , S. Heye , et al., “Transcatheter Arterial Embolization for Iatrogenic Hemobilia: Case Series and Review of the Literature,” European Journal of Gastroenterology & Hepatology 24, no. 8 (2012): 905–909. 22617365 10.1097/MEG.0b013e328354ae1b

[10] D. Ion , C. I. Mavrodin , M. B. Șerban , T. Marinescu , and D. N. Păduraru , “Haemobilia: A Rare Cause of Upper Gastrointestinal Bleeding,” Chirurgia 111, no. 6 (2016): 509–512. 28044954 10.21614/chirurgia.111.6.509

